# Pornography Use Profiles and the Emergence of Sexual Behaviors in Adolescence

**DOI:** 10.1007/s10508-021-02140-3

**Published:** 2021-11-22

**Authors:** Davide Pirrone, Mariëlle Zondervan-Zwijnenburg, Ellen Reitz, Regina J. J. M. van den Eijnden, Tom F. M. ter Bogt

**Affiliations:** 1 Department of Interdisciplinary Social Science, Utrecht University, Padualaan 14, 3584 CH Utrecht, The Netherlands; 2 Department of Methodology and Statistics, Utrecht University, Utrecht, The Netherlands; 3 Department of Clinical Child and Family Studies, Utrecht University, Utrecht, The Netherlands

**Keywords:** Pornography, Sexual behaviors, Young adolescents, Development

## Abstract

This research longitudinally explored adolescent pornography (porn) consumption and its association with sexual development in early and middle adolescence. A four-wave design with half-year intervals investigated pornography consumption and different (sexual) activities, such as masturbation, French kissing, petting, giving/receiving manual and oral sex, and intercourse, among 630 respondents (47.9% female, mean age 13.7 years; SD = 0.48) years at *T*1). A latent growth mixture analysis of pornography consumption revealed two groups with relatively low pornography (LP; 51.8% of the boys, 91.4% of the girls) versus high pornography (HP; 48.2% of the boys; 8.6% of the girls) consumption across time. At *T*1, HP boys on average watched pornography less than once a month, but more than once a year at *T*1. At *T*4, their average pornography use had increased to almost one to two times a week. LP boys never watched pornography at *T*1. At *T*4, their average pornography use was still less than once a year. At *T*1, HP girls never watched pornography, but consumption increased to almost one to three times a month at *T*4. Across waves of the study, LP girls (almost) never watched pornography. A discrete-time survival mixture analysis of sexual developmental patterning indicated that, compared to their LP peers, both girls and boys in the HP groups showed accelerated development of masturbation, petting, and receiving manual sex. Girls in the HP group were also more inclined to receive oral sex, whereas boys in the HP group also showed earlier and more frequent manual sex and intercourse. Thus, whereas the HP group of boys was substantially larger compared to that of girls, pornography consumption was related to accelerated development of sexual activities for both genders across early and middle adolescence. The discussion deliberates on pornography as a driving force in adolescent sexual development versus pornography as a medium of choice for sexually advanced adolescents.

**Supplementary Information:**

The online version contains supplementary material available at 10.1007/s10508-021-02140-3.

## Introduction

In recent decades, pornography has become an integral part of contemporary popular culture. The rapid expansion of the Internet, through which users can now easily access unprecedented amounts of sexually explicit internet material (SEIM) even when they are underage, facilitated the increase in the consumption of porn. A substantial proportion of young people from various countries worldwide indeed report consuming pornography (Alexandraki et al., 2018; Koletić, 2017; Owens et al., 2012; Peter & Valkenburg, 2016). Media plays an important role in the development of adolescent beliefs, attitudes, and behaviors regarding romantic and sexual relations and practices (L’Engle et al., 2006).

Sexual development across adolescence advances from less to more sexually intimate behavior, following a specific trajectory (Cowart-Steckler, 1984; Feldman et al., 1999; Jakobsen, 1997; Lam et al., 2002). Most young people start by holding hands and French kissing, progress to fondling and petting, and subsequently to manual and oral sex and intercourse (De Graaf et al., 2009; Hennessy et al., 2009; Steinberg et al., 2019). Sexy media consumption may accelerate the transition to more intimate sexual behaviors, such as some forms of noncoital sex and intercourse (Brown et al., 2006; Brown & L’Engle, 2009; Collins et al., 2004; Vandenbosch & Eggermont, 2013). To our knowledge, so far, no study has detailed the relations between, in particular, exposure to pornography and this stepwise development of activities across sexual trajectories. Since pornography, as a medium relevant to a substantial number of adolescents, can affect adolescents’ beliefs, attitudes, and behaviors, the study of its consumption in relation to sexual trajectories is timely.

### Explaining the Link Between Media Use and Sexual Behavior: The 3A and Reinforcing Spiral Models

Media can act as a “super-peer” and give normative guidance about sex in addition to what adolescents learn directly from their parents or peers (Brown et al., 2005). Researchers in the tradition of social cognitive theory (SCT; Bandura, 1977, 1986) or cultivation theory (CT; Gerbner et al., 1994) have proposed that extensive exposure to media’s portrayal of ideas, attitudes, and behaviors may encourage adolescents to adopt them and enhance the development of their sexual scripts (Gagnon & Simon, 2005; Wright & Donnerstein, 2014). Defining the script concept further, Wright (2011) developed his 3A model, assuming that voluntary and willful exposition to pornography can lead to the acquisition of behavioral scripts presented in these media, their activation in specific real-life social contexts, and their application in this social context, i.e., a romantic or sexual encounter. In his reinforcing-spiral framework, Slater (2007) posited a reciprocal relationship between media consumption and media effects, implying that pornography use and script acquisition, activation, and application may reinforce each other, particularly if real-life consequences of script application are positive.

Several studies provide empirical evidence that pornography can indeed enhance sexual scripts leading to sexuality tinted behavior. For example, Braithwaite et al. (2015) longitudinally examined the links between watching porn, script acquisition, and sexual risk behavior. They found that students watching pornography more often favored permissive sexual scripts, “hooked up” with sex partners more often, had a higher number of “one night stands,” and planned to have a higher number of sex partners in the future, compared to peers watching pornography less often. In a replication of this study among a younger sample of adolescents, Rasmussen and Bierman (2018) found similar results. When controlling for an important psychological confounder such as risk-taking propensity, adolescents reporting early and regular pornography use tended to initiate sexual activity earlier and indicated to have had nearly double the number of sexual partners compared to peers in a low pornography use trajectory. However, other investigations, among Croatian adolescents, starting from the same theoretical assumptions did not, or only to a limited extent, find effects of pornography use on sexual risk taking—no condom use when having sex, sex under the influence of drugs and alcohol, having multiple partners, etc., —or on enhancing permissive sexual attitudes, implying that porn’s script activation and effects may be specific to the domain under study and the cultural context in which the study is undertaken (Koletić et al., 2019a, 2019b; Matković et al., 2018).

### Motivations, Prevalence, and Relevance of Adolescent Pornography Use

Many adolescents find pornography interesting and exciting. Brown and Bryant (1989) note that young people, for hundreds of years, have turned to the sexually explicit materials of their age to satisfy their curiosity about nude bodies and sexual acts and to arouse themselves. Paul and Shim (2008) listed the motivations of emerging adults to watch pornography and noted that it is consumed by them, first, to manage mood (arouse themselves and masturbate) or as entertainment when bored or depressed. Second, some of them may find it pleasant to fantasize that they interact with the models in the depictions. Third, watching pornography together can help maintain or enhance relations and to become closer socially. Finally, young adults may watch out of habit or because they are addicted to sexually explicit material.

Pornography use is heavily gendered. Much evidence has been brought forward that males are more inclined to seek pornography than females (e.g., Löfgren-Mårtenson & Månsson, 2010; Paul & Shim, 2008; Wright & Štulhofer, 2019). In a recent large Canadian study, Böthe et al. (2020) found similar, relatively early, and high prevalence of pornography consumption among heterosexual, cisgender, and sexual/gender minority boys, and later and lower prevalence among their female peers. However, sexual/minority girls were more inclined to use pornography than heterosexual cisgender girls, owing to sexual orientation-related information seeking. In addition to gender, age and education level are basic variables affecting pornography use. Older adolescents tend to watch pornography more often than younger ones and adolescents with lower academic achievement consume pornography more often than those with higher achievement (De Graaf et al., 2009; Matković et al., 2018). It may seem rather obvious that young adults high on sexual affect watch more pornography than their peers with mitigated sexual desires (Paul & Shim, 2008).

Only a limited number of studies have tried to model pornography use trajectories across adolescence. Doornwaard et al. (2015a) followed a group of Dutch adolescents in grades 8–10 for 2 years and found four trajectories. A substantial group of boys (35%) and the grand majority of girls (92%) reported no or infrequent use; other groups were characterized by occasional, increasing, or decreasing use. Willoughby et al. (2018) assessed a convenience sample of US adults, gathering retrospective data on their pornography use in adolescence and young adulthood. The main distinction they found was between youngster either engaging with pornography or not. Three groups of adolescents were qualified as early, pubescent, and late engagers in viewing porn, while more than half of their sample fell into the group of abstainers, with no or infrequent use. On the basis of a two-wave representative telephone survey of US adolescents, Rasmussen and Bierman (2018) found three trajectories. They distinguished between adolescents in a low-to-moderate pornography use trajectory (38%), an increasing to regular use trajectory (9%), and, again, a large group of low-consumption adolescents (53%) with no use or rare use of pornography. To sum up, across different cultural contexts pornography appears to be a salient medium for at least half of adolescents, more so for boys than girls, and older compared to younger adolescents.

Ward (2003) noted that sexually explicit media presents a rather unrealistic and skewed account of sexuality. Sex is glamorous, fun, and risk-free, and casual sexual interactions are the norm. Safe sex is an exception in the pornographic imagination (Gorman et al., 2010; Grudzen et al., 2009). Some researchers have noted that pornography videos often reflect the wishes and cravings of their dominantly male audience (Barron & Kimmel, 2000; Bridges et al., 2010; Prince, 1990). Other studies have nuanced the observation that women are used merely as instruments for male pleasure (Klaassen & Peter, 2015; McKee, 2005).

We do not yet know much about what, specifically, adolescents watch. Only a few studies have addressed adolescent pornography consumption. VandenBosch (2015) showed that younger adolescents are more likely to consume affection-themed explicit material, while older adolescents more frequently expose themselves to dominance-themed porn. Furthermore, self-defined masculine boys and feminine girls more frequently search for violence-themed material. What adolescents “see” in pornography depends on their interpretation of the material. Adolescents who perceive pornography as “real” and believe that what they see is relevant to their sexual practice are more inclined to adopt its messages (Baams et al., 2015; Peter & Valkenburg, 2010). It has to be noted though, that while use of sexually explicit material increases across adolescence, the evaluation of this material as realistically reflecting sexual practices decreases. In other words, seeing more does *not* make it more realistic (Wright & Štulhofer, 2019).

Still, across subgenre boundaries, the message of pornography is that sex is pleasant, fulfilling, and risk-free. Actors engaging in this activity may present attractive role models. Assuming that adolescents watch voluntary and intentionally, pornography consumption may foster the acquisition of the idea that (casual) sex is exciting and pleasurable and that engaging in a range of sexual activities, from manual and oral sex to vaginal and anal sex, might be worth pursuing. Intimate, porn-derived romantic encounter scripts may be activated and applied.

### Pornography and the Development of Adolescent Sexual Behaviors

Only a small number of studies have addressed the link between pornography consumption and, specifically, sexual behavior. In one of the first studies in this field, Collins et al. (2004) found that 12–17-year-old US adolescents who viewed more television programs with sexual content at baseline were more likely to progress to advanced noncoital sexual activities (kissing, touching, petting, oral sex) and intercourse in the subsequent year. Brown et al. (2006) noticed that 12–14-year-old US white, but not black adolescents, exposing themselves to sexual content in music, movies, television, and magazines, were more often engaged in noncoital sexual activities and sexual intercourse in the following two years compared to those exposed to programs with lighter sexual content. Hennesy et al. (2009) reported a similar finding. Brown and L’Engle (2009) noted that both female and male adolescents, with a mean age of 13.6 years at baseline who consumed adult magazines and explicit internet material, were more likely to have had oral sex and sexual intercourse within the two years following the first measurement. Other researchers have suggested that the relationship is bidirectional, as sexually active adolescents are attracted to erotic media content (Bleakley et al., 2008).

Doornwaard et al. (2015a) followed a group of Dutch adolescents in grades 8–10 with a mean age of 14.3 years at *T*1 for 2 years. Adolescents in groups that did not consume SEIM or consumed it infrequently (35% boys, 92% girls) reported less noncoital and coital activity compared to adolescents in groups in which the exposure to SEIM was either stable, decreased slightly, or increased significantly. Initial levels of sexual activity in non-user groups were lower and remained so across adolescence. An additional analysis of the same Dutch group of adolescents that now included also 7th graders confirmed concurrent links between SEIM use and sexual behavior across adolescence. For boys, but not for girls, SEIM use at *T*1 predicted increases in sexual behavior at *T*2 (Doornwaard et al., 2015b). In another three-wave survey study conducted with 13–17-year-old Dutch adolescents, Vandenbosch and Van Oosten (2017) found that SEIM use enhanced casual sex practices over time. Interestingly, Vandenbosch and Eggermont (2013) noted that among adolescents in the early pubertal stage, consumption of explicit material spurred sexual initiation (coitus), but this relation was negative for adolescents in the advanced pubertal stage, suggesting that early adolescence is an optimal period in which explicit material may be effective. In contrast to the studies listed above, Matković et al. (2018) did not find any indication that the frequency of consuming sexually explicit material use predicted sexual debut in either male or female adolescents, but some evidence for the role of the timing of first exposure to explicit material was present in some of their subsamples. Adolescents in the later exposure to pornography tertile had a significantly lower probability of reporting sexual debut.

### The Present Study

All studies reviewed above contained large samples and strong sets of confounding factors, thus teasing out unique media effects regarding adolescent sexual behaviors. Nearly all of them provide convincing evidence that consuming pornography is relevant for accelerating sexual development and that their effects vary by gender, age, and ethnicity. However, as noted before, sexual development across adolescence is patterned, advancing from less to more sexually intimate behavior (Cowart-Steckler, 1984; Feldman et al., 1999; Hennessy et al., 2009; Jakobsen, 1997; Lam et al., 2002), and in this process, different steps are taken following, more or less, a fixed order. The studies above have analyzed one or two of these steps, such as oral sex or intercourse (Brown & L’Engle, 2009; Vandenbosch & Egermont, 2013; Vandenbosch & Van Oosten, 2017). Alternatively, sum scores of the noncoital activity or the most advanced level of noncoital sexual activity were regarded as outcomes (Brown et al., 2006; Collins et al., 2004; Doornwaard et al., 2015a, 2015b; Hennessy et al., 2009). However, none of these studies have recorded the distinct individual steps adolescents tend to follow in their sexual development. Therefore, this study aimed to, first, identify pornography consumption patterns among adolescents and, second, relate pornography use to the development of the entire trajectory of young people's sexual behavior, from kissing, masturbating, performing, and receiving manual or oral sex to intercourse. This strategy would provide a more detailed account of pornography use and sexual development than the current literature provides.

Gender is a key factor in both media use and sexual behavior. Therefore, we introduced gender as the main distinctive category in our analyses. We aimed to plot different pornography consumption patterns in relation to sexual behavior for girls and boys separately. As pornography consumption and sexual behaviors are related to vocational educational level and advanced pubertal status (De Graaf et al., 2009; Vandenbosch & Eggermont, 2013, 2015), and as it can be assumed that adolescents’ romantic and relational status may be related to pornography use and sexual behaviors as well, we controlled these factors in our analyses.

This leads to the following research questions:

1. Which pornography use trajectories can be distinguished among adolescent girls and boys?
2. Does pornography use relate to advancements in sexual trajectories; more specific, does it accelerate experiencing (a) masturbation, (b) French kissing, (c) petting, (d) performing manual sex, (e) receiving manual sex, (f) performing oral sex, (g) receiving oral sex, (h) vaginal intercourse, and (i) anal intercourse?

Though earlier studies have found a positive link between pornography consumption and increased sexual activity, and it is therefore plausible that pornography use may accelerate sexual development, we refrain from detailed hypotheses regarding the onset of specific behaviors. In that sense, this study was exploratory.

## Method

A detailed html report of all analyses is provided at osf.io/f8bcs.

### Participants and Procedure

Data for this study were collected as part of Project STARS (Studies on Trajectories of Adolescent Relationships and Sexuality), a longitudinal research project on the romantic and sexual development of Dutch adolescents. Adolescents were recruited from four secondary schools in several municipalities throughout the Netherlands. Before the first measurement, both adolescents and their parents received letters, brochures, and flyers describing the study's aims and the option to withhold consent (of their child). Less than 10% of the approached adolescents either decided not to participate, or their parents did not allow them to participate in the study.

Adolescents were followed across four waves, with six-month intervals between waves. The first measurement was conducted in the fall of 2011. At each wave, adolescents completed a computerized questionnaire in their class during regular school hours. Researchers and trained research assistants were present to supervise the data collection, while teachers were not to ensure the confidentiality of responses. Respondents were instructed that they could stop participating at any time.

The longitudinal sample consisted of 668 Project STARS participants (47.8% female). To keep a focus on early adolescence, participants were selected if their age was between 13 and 15 years (*M* = 13.7, SD = 0.48) at *T*1. Missing data on pornography use, sexual experience, and pubertal status were handled with full information maximum likelihood estimation (FIML). Missing data on the (exogenous) categorical control variables could not be dealt with by this method, and these participants were subject to listwise deletion, resulting in a sample of 630 adolescents. Listwise deletion is acceptable when data are missing completely at random (MCAR). Missing data analyses by means of chi-square (for categorical variables) and Kruskal–Wallis (for continuous variables) tests revealed that the 38 participants with missing data on romantic and relational experience did not differ in gender (*p* = .829), age (*p* = .673), or total number of different sexual experiences at *T*2 (*p* = .714), but they did have a lower education level (*p* = .021). As there was no suitable alternative, we continued with listwise deletion for these 38 participants.

Of the 630 remaining participants, 482 (76.5%) contributed data in all four waves. At *T*1, *T*2, *T*3, and *T*4_,_ the number of participants was 612 (97.1%), 582 (92.4%), 565 (89.7%), and 531 (84.3%), respectively. At T1, the 18 missing participants had a lower educational level (*p* < .001), but were comparable to the participants without missing data with respect to their gender, age, and number of different sexual experiences. At *T*2, *T*3, and *T*4, the missing participants more often had a lower education level, higher age, and more different sexual experiences. We assume that these data were missing at random (MAR), for which FIML is a suitable missing data handling method.

The adolescents in the study represented five age cohorts of adolescents in four consecutive grades: the first four years of secondary school (seventh through tenth grade). Most participants (79.4%) had a Dutch background (they and both their parent were born in the Netherlands), 13.8% had a Western background, and 6.8% had a non-Western background (they or a least one of their parents was born in an African, Middle Eastern, Asian, or South-American country). Adolescents were enrolled in different educational tracks, with 49.4% in vocational education programs and 50.6% in college or university preparatory programs.

### Measures

#### Pornography Use

Adolescents’ pornography use was assessed with one item: “How often do you use the Internet to view a pornography Web site (a Web site with pictures or movies that show nudity or people having sex)?” Responses were measured on a 6-point scale ranging from 0 = *Never* to 5 = *Three times a week or more*.

#### Sexual Experience

Participants were first asked, “Have you ever French-kissed somebody?” (0 = *No*, 1 = *Yes*). A two-step question followed, starting with: “Have you ever had sex with another person? With sex, we mean everything from touching or caressing to intercourse” (0 = *No*, 1 = *Yes*). Those who indicated *Yes* received a follow-up question: “With which of the following things do you have experience? You can check multiple boxes.” The options were, naked touching or caressing, performing manual sex, receiving manual sex, performing oral sex, receiving oral sex, vaginal intercourse (penis in vagina), and anal intercourse (penis in anus). The final sexual experience item for all participants was, “Have you ever masturbated?” (0 = *No*, 1 = *Yes*).

#### Control Variables

*Education level* Adolescents’ educational level was operationalized as vocational education (primary school, secondary school VMBO) versus college/university preparatory education (HAVO, HAVO/VWO, VWO, Atheneum, Gymnasium). If a participant switched levels between waves, the most often reported level was used. If a participant scored 50% vocational and 50% college/university preparatory education, the most recent educational level was used as an indicator.

*Pubertal status* Pubertal status was measured by different items for boys and girls. Since puberty starts, on average, one year earlier for girls and this one-year difference persists throughout the development of puberty (Kail & Cavanough, 2010; Mensah et al., 2013), different measurement waves were used for boys and girls. For most items, the response categories were *has not yet started, has barely started, is definitely underway,* and *seems completed.* The items for boys were measured at T4 (spring 2013) and assessed the status of their growth spurt, voice changes, beard growth, ejaculation, and pubic hair growth (confirmatory factor analysis model fit: *χ*^2^(9) = 17.72, *p*-value = .039, CFI = 0.97, TLI = 0.95, RMSEA = 0.06, RMSEA 90%CI = [0.01, 0.10], SRMR = 0.03). The items for girls were measured at *T*2 (spring 2012) and assessed the status of their growth spurt, skin changes, menstruation, breast, and pubic hair growth (confirmatory factor analysis model fit: *χ*^2^(5) = 14.98, *p*-value = .011, CFI = 0.94, TLI 0.87, RMSEA = 0.08, RMSEA 90%CI = [0.04, 0.13], SRMR = 0.04).

*Romantic and relational status* Participants responded to the questions, “Have you ever been in love?” and “Have you ever had a romantic relationship?” (0 = *No*, 1 = *Yes*).

### Analyses

**Fig. 1:**
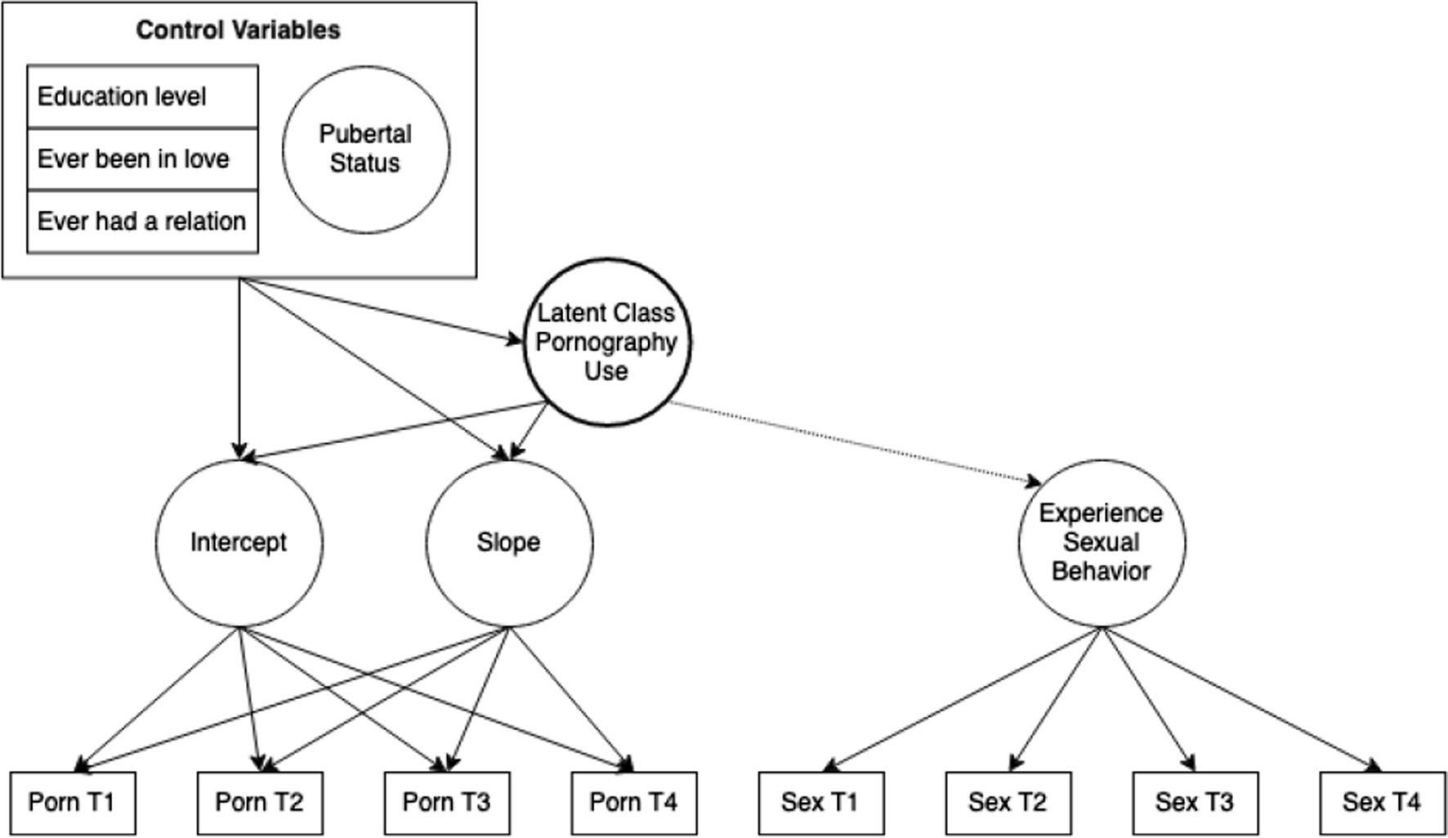
A latent growth analysis mixture model of pornography use and a discrete-time survival mixture model on experience with sexual behavior

First, to answer RQ1 for boys and girls separately, latent growth mixture analyses on pornography consumption were conducted, with education level, pubertal status, and romantic and relational status as continuous and categorical control variables. We started with a one-class linear latent growth mixture model with the intercept at *T*1. Classes were added as long as one of the fit measures indicated that more classes would improve the model and the software did produce errors, which generally indicate underidentification of the model (Nylund et al., 2007). The fit measures under consideration were the Lo–Mendell–Rubin test (LMR), adjusted or Vuong–Lo–Mendell–Rubin test (VLMR), bootstrap likelihood ratio test (BLRT), the Bayesian information criterion (BIC; Tein et al., 2013), and Akaike information criterion (AIC). When fit measures converge on different models, we inspected the different solutions, considering their relations and the stability of the solutions (Nylund et al., 2007). To answer RQ2, we adopted the three-step approach by Vermunt (2010). This method preserves the classification uncertainty from the latent growth mixture model to estimate the distal discrete-time survival models on experience with sexual behavior (Fig. 1) and is therefore recommended over separate classification and analysis (Kamata et al., 2018). As a first step, the latent growth mixture analysis was estimated. As a second step, the most likely class nominations were saved. As a third step, the distal discrete-time survival mixture model was added for each of the sexual experience variables separately. In the third step, the latent class nominations saved in Step 2 were modeled as a nominal covariate. Furthermore, the classification probability logits from step 1 were integrated as fixed classification probability logits for the newly estimated classes. In this manner, the information about classification uncertainties was preserved.

All mixture analyses were executed in Mplus (Version 8.6, Muthén & Muthén, 1998–2006) supported by Mplus Automation (Hallquist & Wiley, 2018) through R (R Core Team, 2020).

## Results

### RQ1: Latent Growth Mixture Analysis

**Fig. 2:**
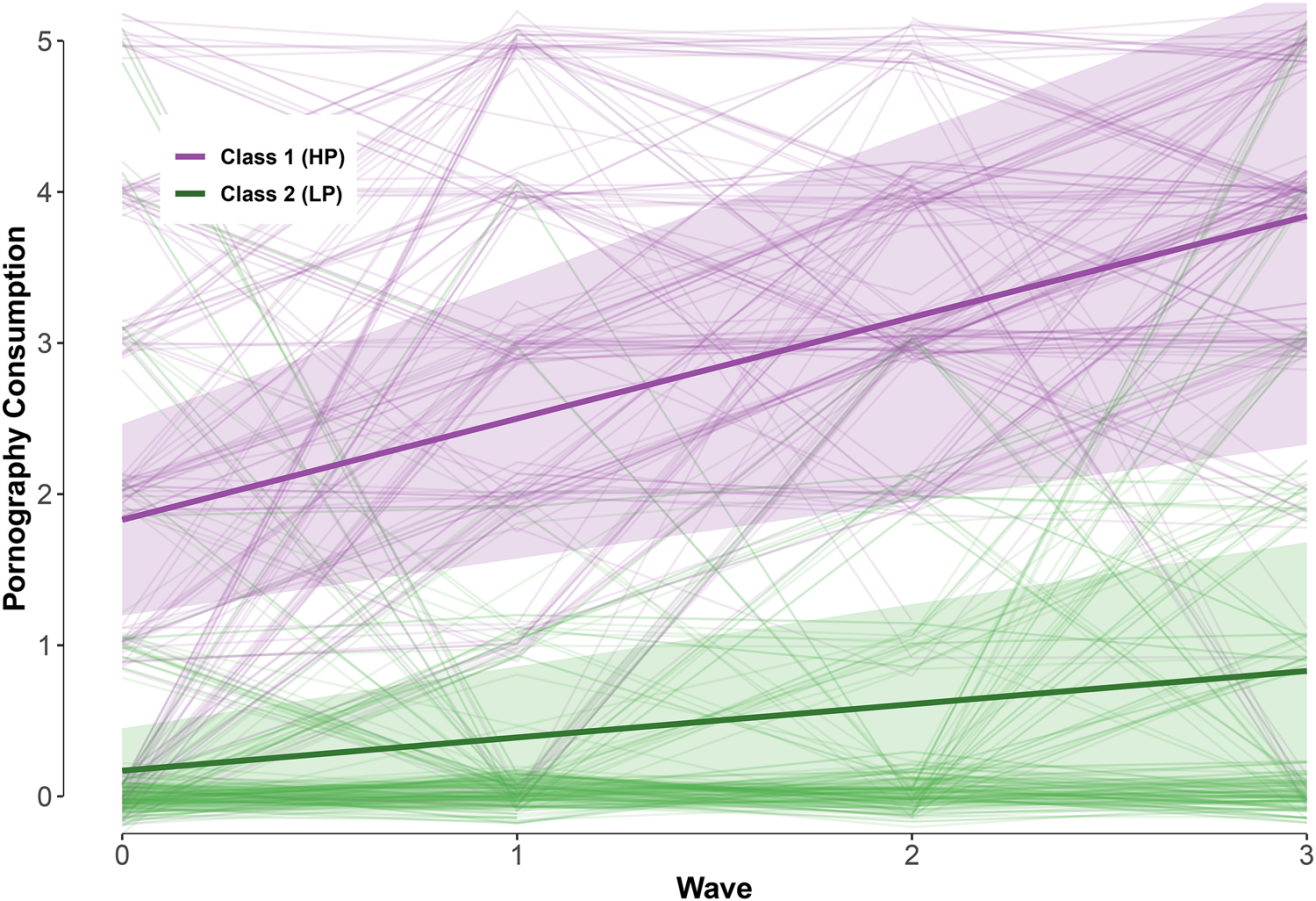
Development (± 1SD) of pornography consumption in a two-group classification of boys; HP versus LP. Class 1 comprises 48.2% of the boys in the sample

*Boys* Table 1 shows the outcomes of the growth mixture models for boys. The associated growth patterns are shown in Figure S1. The four-class solution suffered from estimation issues related to within-class singularity regarding the covariates and the smallest class in this solution consisted of only 12 boys. The small size of the last group obstructed controlling for antecedents, further analysis of consequences of group membership, and the estimation issues made the results unreliable. The AIC and BIC kept decreasing as the number of classes increased. Plotting the AIC and BIC values in an elbow plot did not show a clear point of diminishing returns (see osf html-report). The VLMR, LMR, and BLRT *p*-values all preferred fewer than three classes; hence, the two-class solution was selected with the additional advantage over a three-class solution that it would be easier to interpret once the distal mixtures were added to the model. The two-class solution discerned boys with high versus low initial levels and further development of pornography use (HP versus LP, see Fig. 2). This solution's entropy was 0.83, with 48.2% (*n* = 158) of the boys allocated to the HP class. Table 2 shows the main results of this model. Pubertal status and relationship status significantly predicted the classification. The HP group’s intercept of 1.83 was significantly different from zero and revealed a significant increase over time. In practice, this meant that the HP group on average watched pornography less than once a month, but more than once year at *T*1. At *T*4, their average pornography consumption increased to almost one to two times a week. In the LP group, the intercept and slope did not significantly differ from zero, meaning that on average they (almost) never used the internet to watch pornography throughout the study. At *T*4, however, their average (0.88) was closer to watching pornography *less than once a year* than it was to *never.*

**Table 1:** Latent class fit statistics in boys

Classes	AIC	BIC	Entropy	VLMR *p*-value	LMR *p*-value	BLRT *p*-value	*n*_min_	*n*_max_
1	7558	7683	–	–	–	–	328	328
2	7478	7629	0.832	.091	.097	< .001	158	170
3	7405	7583	0.822	.109	.114	< .001	81	150
4	7331	7536	0.873	.031	.032	.05	12	139

*AIC* Akaike information criterion, *BIC* Bayesian information criterion, *VLMR* Vuong–Lo–Mendell–Rubin likelihood ratio test, *LMR* Lo–Mendell–Rubin likelihood ratio test, *BLRT* bootstrap likelihood ratio test

**Table 2:** Latent class and growth factor estimates for boys

Parameter	High pornography use class			Low pornography use class		
	Est	95% CI	*p*	Est	95% CI	*p*
*Latent class (OR)*						
Education	0.70	[0.30, 1.64]	.411			
Pubertal status	15.85	[1.75, 143.31]	.014			
Ever been in love	3.85	[0.56, 26.34]	.170			
Ever had a relation	3.98	[1.05, 15.17]	.043			
*Intercept factor*						
Intercept	1.83	[1.07, 2.58]	< .001	0.17	[− 0.16,0.50]	.319
Education	− 0.18	[− 0.51, 0.15]	.281			
Pubertal Status	0.74	[− 0.02, 1.49]	.055			
Ever been in love	0.08	[− 0.42, 0.58]	.754			
Ever had a relation	0.40	[− 0.13, 0.93]	.139			
*Slope factor*						
Intercept	0.67	[0.32, 1.01]	< .001	0.22	[− 0.01, 0.44]	.063
Education	0.02	[− 0.14, 0.19]	.776			
Pubertal status	0.06	[− 0.51, 0.63]	.786			
Ever been in love	0.00	[− 0.37,0.49]	.984			
Ever had a relation	− 0.14	[− 0.32,0.03]	.113			
Class count & %	158		48.2	170		51.8

*Girls* Table 3 shows the outcomes of the growth mixture models for girls. Solutions with three and more classes resulted in non-positive definite variance–covariance matrices and singular covariance matrices for the independent variables. The AIC and BIC continued to decrease until the third class with no clear point of diminishing returns. The entropy was high for all class solutions. The (V)LMR *p*-values preferred a one-class solution, whereas the BLRT *p*-value was significant up to three classes. No model was the obvious choice. Hence, we inspected the growth patterns. Figure S2 shows that the one-class solution concerns stable low pornography use. The two-class solution distinguishes a group that starts by never watching pornography and increases somewhat over time, compared to a group that more or less never watches pornography in the course of the study. The three-class solution distinguishes a group that starts higher and decreases somewhat over time, a group that starts negative and increases strongly over time, and a group that is stable at 0. As there could be a meaningful difference between the girls who almost never watch pornography and those regularly watching some pornography, we preferred the two-class solution over the one-class solution. As it is difficult to interpret the three-class solution with a negative intercept, two small classes (*n* = 17 = 5.6% and *n* = 15 ≤ 5.0%), and this solution is also difficult to estimate in combination with distal survival models, we selected the two-class solution.

**Table 3:** Latent class fit statistics in girls

Classes	AIC	BIC	Entropy	VLMR *p*-value	LMR *p*-value	BLRT *p*-value	*n*_min_	*n*_max_
1	4109	4224	–	–	–	–	302	302
2	3473	3618	0.996	0.152	0.156	< 0.001	26	276
3	3090	3265	0.994	0.547	0.553	< 0.001	15	270

*AIC* Akaike information criterion, *BIC* Bayesian information criterion, *VLMR* Vuong–Lo–Mendell–Rubin likelihood ratio test, *LMR* Lo–Mendell–Rubin likelihood ratio test, *BLRT* bootstrap likelihood ratio test

**Fig. 3:**
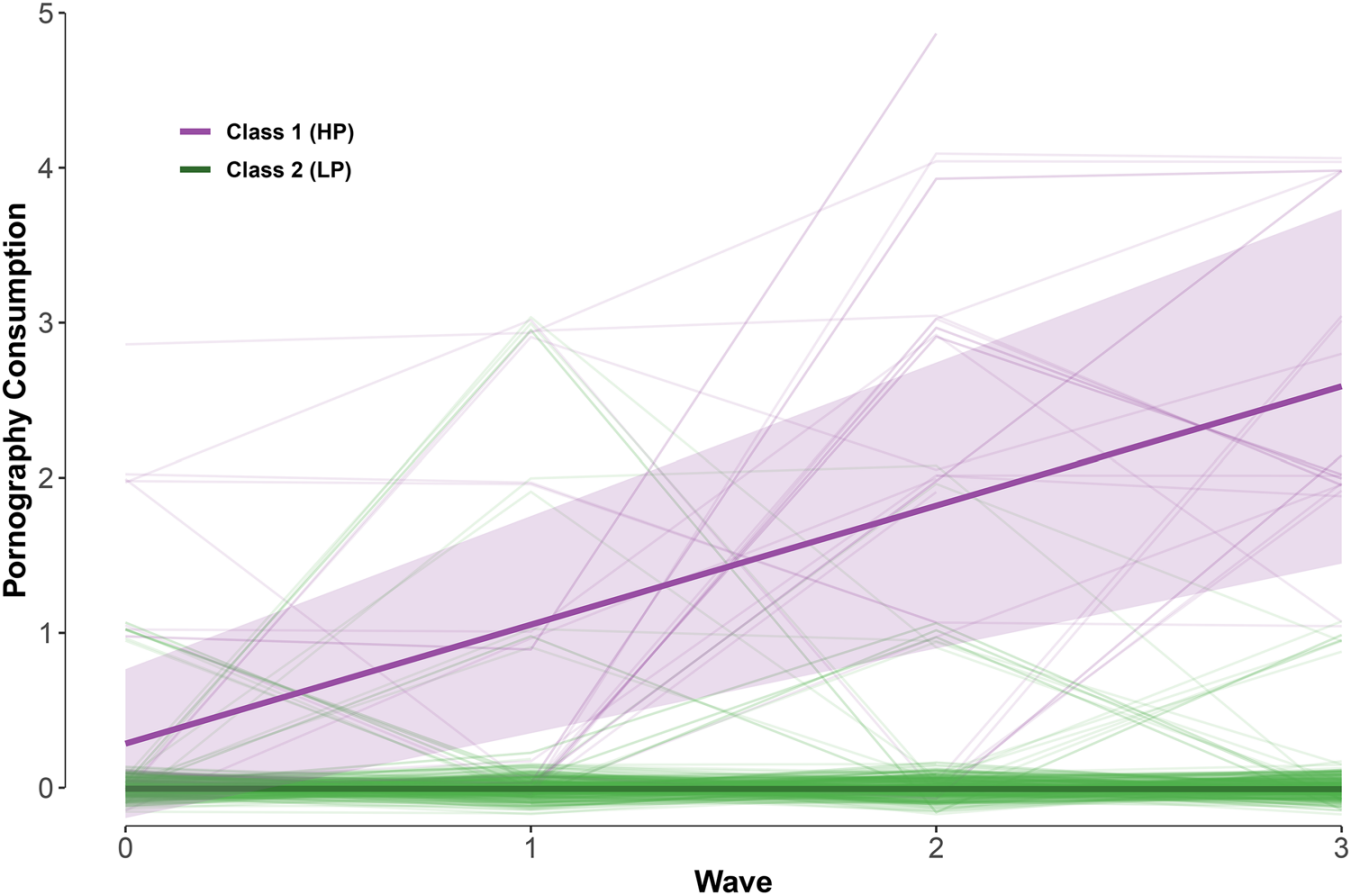
Development (± 1SD) of pornography consumption in a two-group classification of girls; HP versus LP. Class 1 comprises 8.2% of the girls in the sample

The two-class solution discerned girls with no development versus positive development of pornography consumption (HP versus LP, see also Fig. 3). This solution's entropy was 1.00, with 8.7% (*n* = 26) of the girls allocated to the HP group. Table 4 shows the main results of this model. The control variables did not significantly predict the classification. For both the HP and LP groups, the intercept did not significantly differ from zero. However, the linear slope in the HP group showed a significant increase over time, reaching an average of 2.6 at *T*4, which lies in between watching pornography *less than once a month* and *one to three times a month.* In the LP group, the slope did not differ significantly from zero, indicating that the LP group never watched pornography (on average) throughout the study.

**Table 4:** Latent class and growth factor estimates for girls

Parameter	High pornography use class			Low pornography use class		
	Est	95% CI	*p*	Est	95% CI	*p*
*Latent class (OR)*						
Education	0.44	[0.19, 1.09]	.055			
Pubertal Status	3.22	[0.96, 10.79]	.058			
Ever been in love	3.62	[0.46, 28.48]	.221			
Ever had a relation	0.78	[0.31, 2.00]	.605			
*Intercept factor*						
Intercept	0.29	[− 0.29, 0.86]	.328	− 0.01	[− 0.04, 0.03]	.763
Education	0.01	[− 0.03, 0.05]	.615			
Pubertal Status	0.06	[− 0.02, 0.14]	.161			
Ever been in love	0.04	[− 0.00, 0.07]	.058			
Ever had a relation	0.00	[− 0.05, 0.05]	.998			
*Slope factor*						
Intercept	0.77	[0.51, 1.03]	< .001	− 0.00	[− 0.04, 0.03]	.974
Education	− 0.00	[− 0.03, 0.03]	.880			
Pubertal status	− 0.05	[− 0.09, − 0.00]	.041			
Ever been in love	− 0.01	[− 0.03, 0.02]	.734			
Ever had a relation	0.01	[− 0.03, 0.04]	.619			
Class count & %	26		8.7	276		91.4

### RQ2: Sexual Experiences

**Fig. 4:**
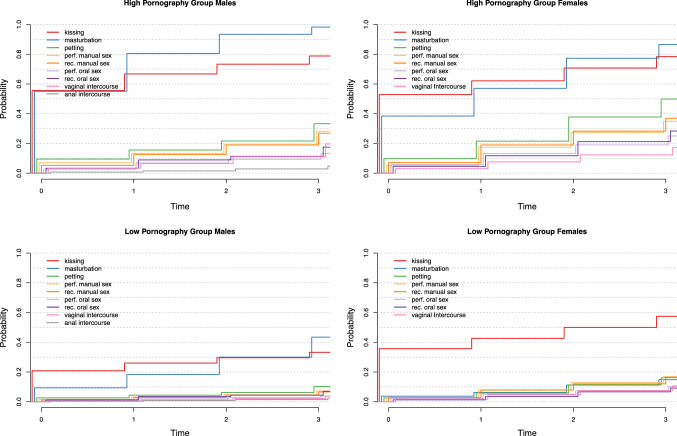
Sexual behavior profiles for boys and girls, high and low in pornography consumption

Figure 4 shows the profiles (i.e., experience with all investigated sexual behaviors) for boys and girls in the LP and HP groups. We could not statistically compare the different behaviors as they were part of different models, but we could discern patterns visually. For example, masturbation and kissing were particularly elevated for both boys and girls in the HP groups compared to the LP groups. Moreover, in HP boys, the probabilities of having experience with petting and manual sex (performing and receiving) were similar over time, as did experience with oral sex (performing and receiving) and vaginal intercourse. Although experience with masturbation and kissing was common in LP boys, experience with other forms of sexual behavior was rare. In HP girls, the probability of having experience with sexual behaviors varied per type of sexual behavior; only the probabilities of performing and receiving manual sex, and the probabilities of performing and receiving oral sex were strongly connected at each time point. In LP girls, kissing was quite common, but all other forms of sexual behavior were rare, even masturbation. Moreover, the probabilities of having experience with petting and manual sex (performing and receiving) seemed similar over time, as did the probabilities of having oral sex (performing and receiving) and vaginal intercourse.

The discrete-time survival mixture model demonstrated the temporal association of pornography consumption with experiencing different sexual behaviors. Figures S3 to S11 show that the HP groups, both boys and girls, displayed higher probabilities of having experienced or performed specific sexual behaviors. The vertical steps in a line show the increased probability of individuals within that group to engage in a certain sexual behavior at that time point. Increases in the probability of sexual behaviors always appear larger at each time point for members of the HP group compared to the LP group (Figures S3–S11). Table 5 shows the predicted results per wave for boys and girls in the LP and HP groups and the total group. For anal sex, we could not run our analysis for girls as none of them engaged in anal intercourse. For each sexual behavior, the odds ratios for the HP group, as compared to the LP group to engage in the sexual behavior of interest throughout the study, are provided in Tables 6 and 7.

**Table 5:** Means for pornography use and % of participants who performed a sexual behavior in total and low versus high pornography consumption groups by wave

Pornography use group	*T*_1_			*T*_2_			*T*_3_			*T*_4_		
	Total	Low	High	Total	Low	High	Total	Low	High	Total	Low	High
Boys												
Pornography use mean (SD)	1.27 (1.77)	0.40 (1.40)	2.20 (1.39)	1.61 (1.69)	0.54 (1.19)	2.74 (1.19)	1.96 (1.71)	0.67 (0.97)	3.29 (0.97)	2.31 (1.98)	0.81 (1.42)	3.84 (1.42)
Sexual behavior (%)												
Masturbation	32.5	10.0	56.3	50.2	19.7	81.9	62.8	31.3	93.9	71.1	43.9	98.3
French kissing	38.4	20.0	58.1	46.3	24.9	69.3	51.1	28.2	75.5	55.7	31.8	81.1
Petting	6.4	2.7	10.47	10.5	4.5	17.0	14.7	6.4	23.6	22.5	10.4	35.6
Performing manual sex	4.6	1.7	7.6	8.7	3.3	14.5	12.1	4.8	20.2	17.8	7.3	29.4
Receiving manual sex	3.4	1.2	5.6	7.8	2.9	13.1	11.9	4.6	20.0	16.8	6.7	28.0
Performing oral sex	1.8	0.8	2.9	4.6	2.1	7.4	6.4	2.9	10.2	8.6	4.0	13.8
Receiving oral sex	2.4	1.3	3.6	6.5	3.6	9.7	8.3	4.6	12.3	12.4	7.1	18.5
Vaginal intercourse	2.1	0.6	3.7	4.0	1.2	7.1	6.9	2.1	12.0	11.8	3.9	20.7
Anal intercourse	0.6	0.4	0.8	1.2	0.8	1.7	2.3	1.5	3.2	3.8	2.5	5.3
Girls												
Pornography use mean (SD)	0.08 (0.35)	0.03 (0.16)	0.32 (0.96)	0.14 (0.61)	0.03 (0.50)	1.09 (0.50)	0.20 (0.72)	0.03 (0.48)	1.86 (0.48)	0.26 (0.78)	0.03 (0.32)	2.63 (0.32)
Sexual behavior (%)												
Masturbation	7.0	4.0	38.6	11.3	6.6	57.3	18.0	11.8	77.4	21.7	15.2	86.1
French kissing	38.4	35.7	53.8	45.1	43.5	62.4	52.1	50.4	70.6	59.5	57.7	78.1
Petting	3.3	2.5	9.9	7.4	6.2	21.5	14.1	12.0	37.8	19.8	17.0	49.8
Performing manual sex	2.6	2.3	5.5	8.5	7.7	16.9	14.0	12.9	27.1	18.5	17.1	34.9
Receiving manual sex	3.3	2.8	7.3	8.8	7.9	18.6	13.7	12.3	28.1	18.5	16.8	36.9
Performing oral sex	2.6	2.2	6.1	5.7	5.0	12.8	8.5	7.5	18.9	11.6	10.2	25.1
Receiving oral sex	1.7	1.3	4.5	4.4	3.7	11.7	8.2	7.0	21.3	11.3	9.6	28.4
Vaginal intercourse	2.0	1.8	3.0	5.1	4.6	7.6	8.2	7.9	12.3	11.6	11.1	17.2

**Table 6:** Sexual experience odds ratio’s for HP as compared to LP groups in boys throughout the study

Variable	Est	95% CI	*p*
Masturbation	11.63	[7.42, 18.23]	< .001
French kissing	5.56	[3.60, 8.58]	< .001
Petting	4.20	[2.17, 8.15]	< .001
Performing manual sex	4.75	[2.22, 10.18]	< .001
Receiving manual sex	4.89	[2.23, 10.77]	< .001
Performing oral sex	3.70	[1.32, 10.38]	.013
Receiving oral sex	2.85	[1.28, 6.33]	.010
Vaginal sex	6.08	[2.11, 17.51]	.001
Anal sex	2.19	[0.55, 8.78]	.268

**Table 7:** Sexual experience odds ratio’s for HP as compared to LP groups in girls throughout the study

Variable	Est	95% CI	*p*
Masturbation	15.27	[8.95, 26.08]	< .001
French kissing	1.99	[1.05, 3.75]	.034
Petting	3.99	[2.04, 7.78]	< .001
Performing manual Sex	2.37	[1.07, 5.24]	.034
Receiving manual Sex	2.60	[1.25, 5.38]	.010
Performing oral sex	2.71	[1.10, 6.69]	.030
Receiving oral sex	3.40	[1.45, 7.95]	.005
Vaginal sex	1.61	[0.55, 4.68]	.381

*Boys.* Concerning masturbation, French kissing, petting, performing manual sex, receiving manual sex, and vaginal sex, boys who belonged to the HP group were more likely to experience these sexual behaviors compared to their LP peers (Table 6). Differences in performing and receiving oral sex were also significant. There was no difference in anal sex (Table 6).

*Girls.* Concerning masturbation, petting, and receiving oral sex, girls who belonged to the HP group were more likely to experience these sexual behaviors compared to the girls in the LP group (Table 7). Differences in French kissing, performing manual sex, receiving manual sex, and performing oral sex were also significant. Having vaginal sex, however, did not significantly differ between the two groups (Table 7).

## Discussion

Although much work has been done on the role of media in developing sexual behaviors (e.g., Hennessy et al., 2009; Vanderbosch & Eggermont, 2013), earlier studies did not chart all the distinctive individual steps in the trajectory of adolescent sexual development. The current study was in the unique position to narrow this gap by identifying pornography consumption patterns and examining their co-occurring engagement in a broad spectrum of sexual behaviors. Our findings revealed two trajectories of pornography consumption for both boys and girls labeled as high pornography use (HP) and low pornography use (LP), although overall higher levels of pornography consumption were found in boys compared to girls. Moreover, adolescents in the two pornography use trajectories differed in their engagement in a broad spectrum of sexual behaviors, both at the initial level and over time.

### Pornography Use Trajectories

Almost half of the boys were in the HP class trajectory (48, 2%). Their average pornography use increased from less than once a month, but more than once a year at *T*1, to almost one or two times a week at *T*4. Their sexual curiosity, which is common during adolescence, seemed to translate into an increased interest in exploring sexuality through pornography (Savin-Williams & Diamond, 2004). However, the other half of the boys (51, 8%) were in the LP class. They, on average, (almost) never used the Internet to watch pornography (although their average at *T*4 indicated at least some porn consumption, but still less than once a year. This indicates that regular pornography use is by no means common among boys. LP boys’ sexual interest was lower than that of the HP boys, or they might express it differently, for instance, by increased fantasizing about sexual acts (Person et al., 1989).

Similar HP versus LP trajectories were found for girls (HP vs. LP), but their proportions differed from boys. Among HP girls average porn watching increased from (almost) never at *T*1 to a level between less than once a month and one to three times a month at *T*4. The large majority of girls (91.8%) in the LP class trajectory (almost) never watched pornography for the duration of the study. Despite comparable increases over time, the initial levels of pornography use were lower among HP girls compared to HP boys. These findings are in accordance with previous studies, indicating that pornography is more attractive to boys than to girls (Peter & Valkenburg, 2006; Bleakley et al., 2011). Several explanations for gender differences in pornography use have been proposed: pornography may portray sexuality in a way that does not attract girls (e.g., made by men for men), girls may prefer less explicit or less visual sexualized content, or shame or guilt may cause them to underreport their actual pornography use (Löfgren-Mårtenson & Månsson, 2010; Byers & Shaughnessy, 2014).

Moreover, at this age, girls’ level of sexual interest may be somewhat lower than that of boys. As the meta-analysis by Petersen and Hyde (2010) showed, boys in general report more sexual behavior compared to girls, and boys’ reports are particularly higher for solitary sexual behaviors, such as masturbation. Nevertheless, the presence of an HP class for girls suggests that some girls are interested in online visuals of sexual behaviors and do not perceive social barriers to consume pornography.

### Sexual Experiences

Our findings demonstrated that adolescents in different trajectories of pornography use show different patterns of development in sexual behaviors. Specifically, while an overall tendency to increase engagement in sexual behaviors exists, boys and girls in the HP classes showed a higher engagement in sexual activities over time than their same-sex peers in the LP classes.

While both HP groups (boys and girls) showed an accelerated sexual development compared to both LP groups, the findings regarding specific sexual behaviors were not always equal for boys and girls in the HP groups. The results suggest that for a substantial group of boys, pornography use is present relatively early in adolescence. Additionally, their pornography use coincides with first sexual experiences, particularly masturbation and French kissing. Next, both pornography use and engagement in different sexual behaviors tend to increase, suggesting that these HP boys go through a sexually active period. On the one hand, pornography use may stimulate both solitary and partner-oriented sexual activities, while on the other hand, adolescents’ increased engagement in solitary- and partner-oriented sexual acts may promote increased levels of pornography consumption. These parallel developments in pornography use and sexual behaviors are consistent with the assumption of the reinforcing-spiral framework (Slater, 2007), according to which pornography use and sexual behaviors may reinforce each other over time.

Different from boys, girls in the HP trajectory did not show a substantially higher initial level of pornography use, but they did show a clear increase in pornography consumption over time. Furthermore, compared to same-sex peers in the LP group, the HP girls showed a higher engagement in specific sexual behaviors, particularly masturbation, petting, and receiving oral sex. No differences were found for vaginal intercourse. A reason that the HP and LP girls do not differ in vaginal intercourse might be that prevalence rates for this type of sexual behavior are low among early adolescents and the adolescent girls included in this study are simply too young (*M* = 13.7 at *T*1). A Dutch epidemiological study, for instance, showed that 50% of adolescents engaged in vaginal intercourse at the age of 18.0 years old, and almost 60% of the girls who did not report to have had sexual intercourse still considered themselves to be “too young” for intercourse (De Graaf et al., 2017).

We observed more robust associations with specific sexual behaviors for both boys and girls in the HP groups compared to the LP groups (e.g., masturbation and petting), perhaps because pornography leads to the acquisition of sexual scripts that may be activated in specific real-life social contexts and that may be applied in a specific social context (e.g., a romantic or sexual encounter), which is consistent with the 3A-model (Wright, 2011). Another explanation, however, would be that both pornography use and these sexual behaviors reflect a relative stronger sexual interest or desire (Paul & Shim, 2008). In addition, the relatively strong relationship between masturbation and pornography consumption may result from the fact that pornography is often used for masturbatory purposes (Miller et al., 2019).

### Strengths, Limitations, and Future Research

The present study has some notable strengths. First, the study used four-wave longitudinal data from a sample of early adolescents and examined relations between pornography use and sexual behaviors from a developmental perspective. By using such an approach, as opposed to a traditional cross-sectional approach, we examined changes in pornography use and sexual behaviors over time while including important control variables, such as pubertal timing and romantic and relational status. Second, research on pornography use is scarce and tends to focus mainly on males. Our study also considered girls’ pornography use; therefore, it provides a more inclusive view of developments in pornography use and co-occurring sexual behaviors in this age group. Third, the study included early adolescents, a group that is rarely included in research on pornography. Early adolescence is a sensitive period during which adolescents are unfolding their sexual behaviors and interests, and it appears to be the most influential period for sexual media to impact individuals’ sexual development (Vandenbosch & Eggermont, 2013). Connected to the frequency of pornography use, respondents indeed showed different transitions into diverse intimate sexual behaviors during this age period. By providing an account of (early) adolescent pornography use in co-occurrence with a differentiated set of steps in sexual development, the present study’s results make a unique contribution to the literature. Nevertheless, several limitations should be mentioned as well. First, the use of self-reported data on pornography usage and sexual behaviors may have yielded biased responses. However, self-reports are inevitable when evaluating pornography use and experiences with sexual behaviors due to the private nature of these sexual behaviors. Second, only one item was used to assess pornography use, limiting our ability to understand the context and content of pornography use. For instance, we could not assess whether pornography in general or pornography depicting specific sexual acts affects the development of certain sexual behaviors. Future research should try to take account of the content and context of pornography use to better understand the complex processes underlying pornography consumption and its effects. Third, the frequency of engaging in sexual behaviors or the number of partners that were involved in these sexual behaviors (e.g., having sex with a partner in a stable relationship vs. having multiple partners frequently) were not taken into consideration because the primary focus of the present study was on trajectories of different types of sexual behavior and how pornography use relates to these trajectories. However, future studies might want to include such factors to give more insight into the contexts in which sexual behaviors take place. Fourth, because of the low age of the sample (*M* = 13.7 at *T*1), the prevalence of certain sexual behaviors, such as vaginal and anal sexual intercourse, was low, limiting the possibility of finding significant differences between HP and LP groups. Fifth, even though we used a longitudinal design, causal inferences cannot be made. Thus, it is unclear whether (early) engagement in sexual behaviors resulted from pornography use, whether increased interest in pornography resulted from engagement in specific sexual behaviors, or whether a generally high level of sexual desire was underlying both behavioral domains. Young adults, for example, who scored high on sexual affect—a person’s overall tendency toward external sexual cues—watched more pornography (Paul & Shim, 2008). Although our analyses controlled for pubertal timing and romantic and relational status, all proxies of sexual interest, and thereby decreased the chance that a third factor, such as sexual desire, influenced the present findings, the possibility of such a third factor affecting sexual activities remains. Therefore, future research should account for a broader range of possible underlying factors, such as sexual preoccupation or depression, as they relate to compulsive use of pornography (Doornwaard et al., 2016). In addition, religiousness may be a factor that decreases intentional expose to porn, as religiously inspired adolescents may restrain themselves because of their restrictive attitudes toward porn, and immersion in religious communities with higher levels of social control (Hardy et al., 2013). Pornography use generally increases across adolescence into young adulthood, but religiousness and religious involvement in local congregations may obscure these increases (Rasmussen & Bierman, 2016). It may be worthwhile for future research to include religiousness as a control variable as well. Finally, our results were based on a convenience sample in the Netherlands, a country often described as open and liberal toward adolescent sexuality (Schalet, 2011). Consequently, the study requires cross-cultural validation, especially in countries with different attitudes toward sexuality.

### Societal Relevance

The present results may inform policymakers, sex educators, and sexual health promoters. First, the results indicate that pornography is popular among a substantial group of adolescents, particularly boys, and that its consumption may affect both boys’ and girls’ sexual behaviors. But about half of adolescent boys and a large majority of their female peers do not report much interest in pornography. This finding is important when looking for the right tone and content of educational messages, when media literacy, sex education, or health promotion are at stake. Second, the findings suggest that sexual media literacy may be particularly important for adolescents who use pornography on a regular basis. Our results indicate that pornography consumption is related to accelerated development of sexual activities, and previous research has shown that early sexual debut can increase the risk of teen pregnancies, teen births, and having an abortion (Heywood et al., 2015). Thus, health promotion messages seem to be particularly important to early adolescents who tend to use pornography. A second risk of (early) pornography use lies in the fact that pornography often portrays an unrealistic, glamorized picture of sexuality. Adolescents may easily accept these unrealistic representations of sexuality as they lack sexual experience. These representations may also affect adolescents who do not even watch (much) porn themselves. For example, girls may feel pressured to adopt sexual scripts present in porn as they believe that their sexual partners expect them to behave like the women they have seen in pornography (Doornwaard et al., 2017). Accordingly, both boys and girls should be educated that pornography does not reflect real-life sex, and sexual education should focus on stimulating adolescents’ personal search for sexual preferences, instead of acting upon the sexual scripts provided by porn. Sex education should preferably be taught before regular pornography use has developed, as it may be assumed that early developed sexual scenarios will persist into adulthood.

### Conclusion

Media are an important resource affecting the development of adolescents’ beliefs, attitudes, and behaviors regarding romantic and sexual relations and practices (L’Engle et al., 2006). Early adolescents may consume pornographic material to learn about sex (Albury, 2014). Our findings corroborate that sexy media matter for many young adolescents and more so for boys compared to girls. Today, the Internet occupies a particularly prominent role in youths’ daily lives. The current study tested an integrative model that identified two levels of pornography use among adolescents (HP and LP for both girls and boys), with higher levels of pornography use being related to accelerated development of sexual trajectories. While the proportion of boys in the HP group was far larger than that of girls, our findings point out that pornography consumption may affect boys and girls similarly (Peter & Valkenburg, 2009). The results suggest reciprocal relationships between pornography use and sexual behaviors, consistent with Wright’s 3A model and the assumption of Slater’s reinforcing-spiral framework, which proposes that adolescents may practice what they have seen and learned and that pornography use and sexual behaviors may reinforce each other over time.

## Supplementary Information

Below is the link to the electronic supplementary material.

Supplementary file1 (DOCX 1993 KB)

## Data Availability

Data have been deposited to Data Archiving and Networked Services (https://doi.org/10.17026/dans-z6b-t8ft).
