# Supplementary material for: Pornography Use Profiles and the Emergence of Sexual Behaviors in Adolescence
Source: Arch Sex Behav. 2021 Nov 22;51(2):1141–56. doi: 10.1007/s10508-021-02140-3 (PMC8888502; doi:10.1007/s10508-021-02140-3)
Supplement: Supplementary file 1 — Supplementary file1 (DOCX 1993 KB) [file 10508_2021_2140_MOESM1_ESM.docx]

**Supplementary Materials
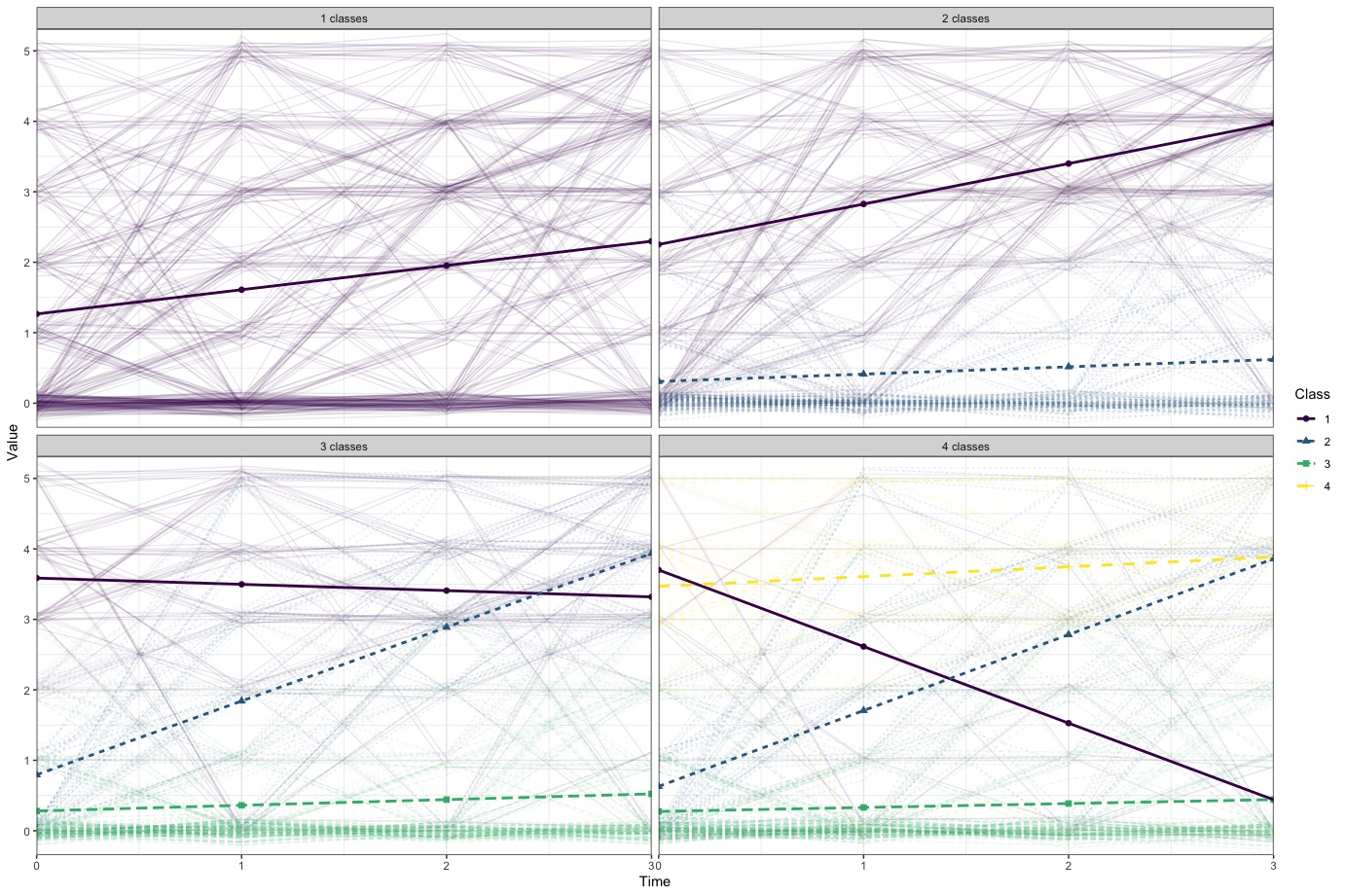
**

Figure S1. Growth patterns for 1-4 latent classes in boys, with individual data plotted in the background.


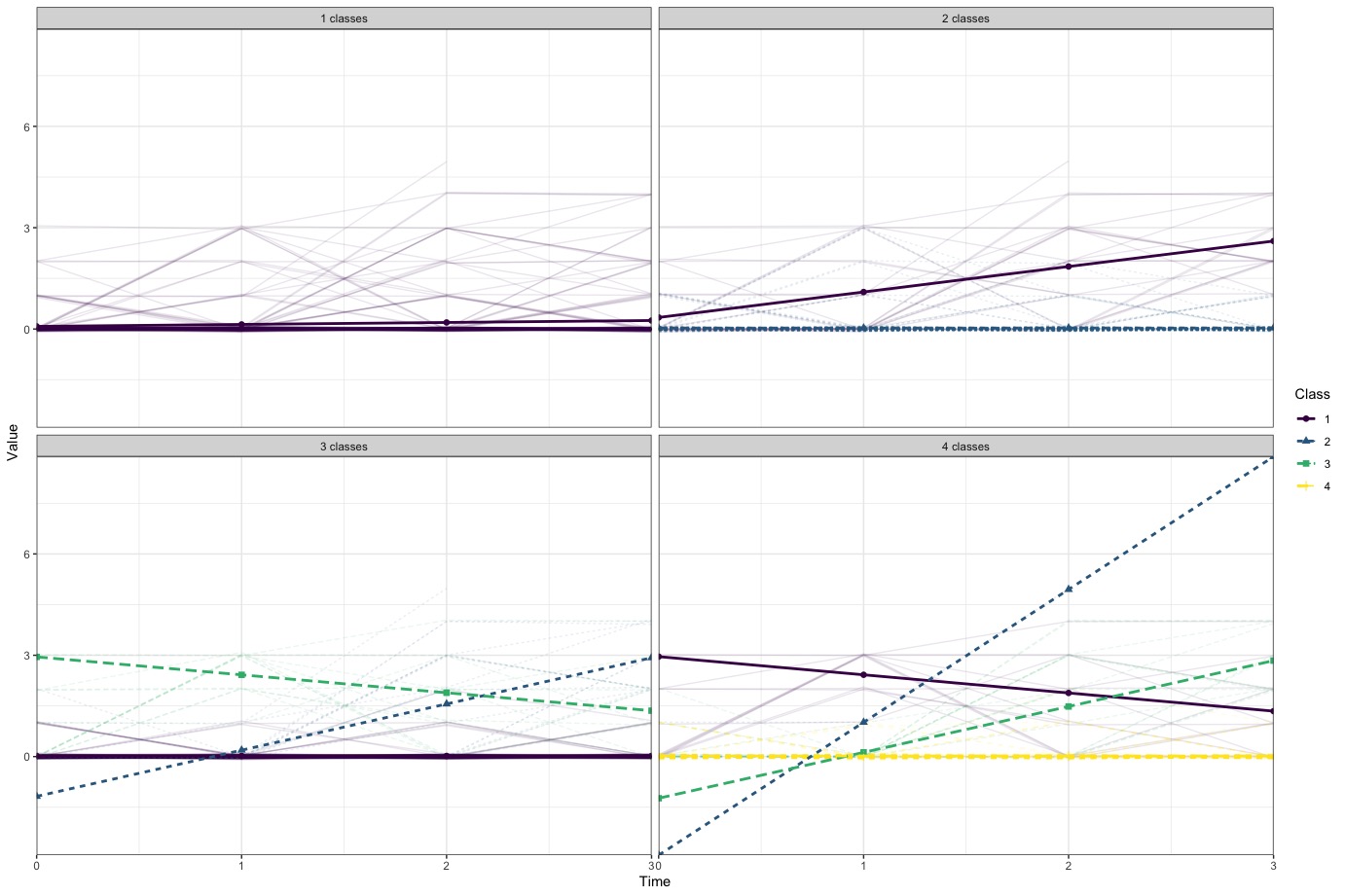


Figure S2. Growth patterns for 1-4 latent classes in girls, with individual data plotted in the background.

*Figure* *S3.* Probability increases in masturbation for boys and girls in the HP and LP classes.

*Figure S4.* Probability increases in kissing for boys and girls in the HP and LP classes..


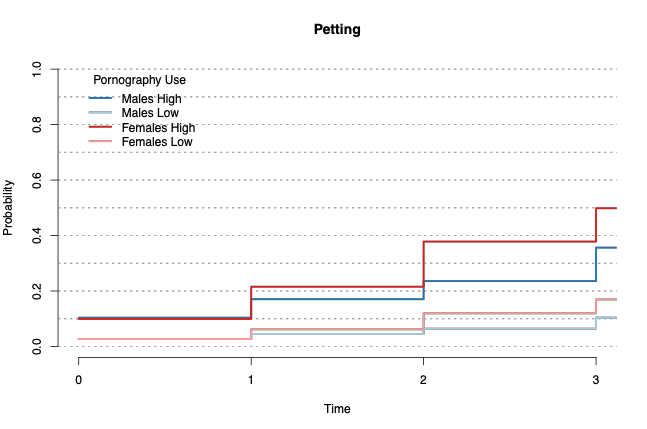


*Figure S5.* Probability increases in petting for boys and girls in the HP and LP classes.

*Figure S6.* Probability increases in performing manual sex for boys and girls in the HP and LP classes.

*Figure S7.* Probability increases in receiving manual sex for boys and girls in the HP and LP classes.

*Figure S8.* Probability increases in performing oral sex for boys and girls in the HP and LP classes.

*Figure S9.* Probability increases in receiving oral sex for boys and girls in the HP and LP classes.

*Figure S10.* Probability increases in vaginal intercourse for boys and girls in the HP and LP classes.

.

*Figure S11.* Probability increases in anal intercourse for boys and girls in the HP and LP classes.
